# Beyond protein modification: the rise of non-canonical ADP-ribosylation

**DOI:** 10.1042/BCJ20210280

**Published:** 2022-02-17

**Authors:** Marion Schuller, Ivan Ahel

**Affiliations:** Sir William Dunn School of Pathology, University of Oxford, Oxford OX1 3RE, U.K.

**Keywords:** ADP-ribosylation, nucleic acids, PARP, protein modification

## Abstract

ADP-ribosylation has primarily been known as post-translational modification of proteins. As signalling strategy conserved in all domains of life, it modulates substrate activity, localisation, stability or interactions, thereby regulating a variety of cellular processes and microbial pathogenicity. Yet over the last years, there is increasing evidence of non-canonical forms of ADP-ribosylation that are catalysed by certain members of the ADP-ribosyltransferase family and go beyond traditional protein ADP-ribosylation signalling. New macromolecular targets such as nucleic acids and new ADP-ribose derivatives have been established, notably extending the repertoire of ADP-ribosylation signalling. Based on the physiological relevance known so far, non-canonical ADP-ribosylation deserves its recognition next to the traditional protein ADP-ribosylation modification and which we therefore review in the following.

ADP-ribosylation is a multifaceted modification of macromolecules that regulates a variety of cellular processes ranging from DNA damage repair, chromatin and telomere-related dynamics, RNA biogenesis, to stress and immune responses including antiviral defence as well as microbial metabolism, pathogenicity and nitrogen fixation [1–6]. The diverse enzyme superfamily of ADP-ribosyltransferases (ARTs) catalyses the ADP-ribosylation reaction which is characterised by the transfer of ADP-ribose from nicotinamide adenine dinucleotide (NAD^+^) onto target substrates via *N*-, *O*-, or *S*-glycosidic linkages with concomitant release of nicotinamide. While the majority of ARTs transfer only single ADP-ribose units onto their targets [7], resulting in substrate mono-ADP-ribosylation (MARylation), a subset of eukaryotic ARTs is also capable of repeatedly attaching an ADP-ribose unit to the respective preceding one. The latter process is known as poly-ADP-ribosylation (PARylation) forming long poly-ADP-ribose (PAR) chains reaching up to 200 units with occasional branching [8,9]. Whether ADP-ribose is attached as monomer or as linear or branched oligomers, influences the outcome for following downstream processes [10–12]. The structural heterogeneity and characteristic negative charge of PAR can additionally alter the properties of the substrate, thus providing another means of target modification, and affects the biophysical properties of local subcellular environments, e.g. by regulating phase separating processes during DNA repair and stress granule formation [13,14].

ADP-ribosylation has traditionally been considered as a post-translational modification (PTM) of proteins. All started almost 60 years ago with the identification of a polymer of ADP-ribose by Pierre Chambon and colleagues which was initially mistaken for a poly(A) reaction product while studying the RNA synthesis by RNA polymerase [15,16]. Following the observation of enzymes being present in mammalian cell extracts that can generate ADP-ribose polymers from NAD^+^ [17], bacterial toxins were then identified to cause pathogenicity through their function as ARTs. Diphtheria toxin, produced by *Corynebacterium diphtheriae*, was among the first to be characterised and found to ADP-ribosylate the eukaryotic elongation factor 2 (eEF2), which results in inhibition of protein biosynthesis and consequently toxicity [18,19]. Studies on cholera toxin isolated from *Vibrio cholerae* followed [20] which was shown to ADP-ribosylate a specific arginine in the regulatory subunit (G_s_α) of heterotrimeric G-proteins that controls adenylate cyclase function, thus leading to unregulated production of cAMP. The elevated cAMP levels activate protein kinase A which opens normally gated channels in the plasma membrane, which results in the profuse watery diarrhea characteristic for cholera pathogenesis [21–24]. These beginnings of the ART research field defined the ADP-ribosylation of proteins as the canonical reaction of ARTs to this day. Furthermore, from those first discoveries research on ARTs and the ADP-ribosylation system expanded immensely over the next decades. ARTs were studied by phylogenetic analyses, which identified these enzymes in all domains of life and some viruses [25], and by functional and structural analyses, revealing enzymatic mechanistic details, target substrates and specificities with identification of exact modification sites. The latter benefitted greatly from proteomics approaches which confirmed the early known glutamate, aspartate, arginine and diphthamide (a modified histidine) residues as ADP-ribosylation acceptors but also identified as such serine, histidine, threonine, tyrosine, lysine and cysteine [7,26–28]. Serine residues were thereby found to be the most common ADP-ribosylation amino acid targets in human cells [29–32].

ADP-ribosylation signalling is regulated by a dynamic interplay between ARTs and ADP-ribosylhydrolase enzymes which are able to reverse the reaction of the transferases by removing the ADP-ribose modifications [1,33–35]. The ART superfamily consists of more than 20 families and its members can be grouped (based on homology of their catalytic domain to the first characterised bacterial ART toxins) into the diphtheria toxin-like ADP-ribosyltransferases (ARTDs) and cholera toxin-like ADP-ribosyltransferases (ARTCs). Four ARTCs are expressed in humans which are extracellular and partly membrane-associated or secreted proteins [27,36]. Most of the eukaryotic members of the ARTD family are referred to as PARPs which form the largest subfamily of ARTs with seventeen members in humans. PARP1, the enzyme involved in DNA repair and chromatin regulation, is the founding member of PARPs, the best characterised so far as well as the also most ubiquitous expressed and abundant PARP [37,38]. Amongst the PARPs, PARP1, 2 and the tankyrases (TNKS1 and 2) show PARylation activity, while all others, with the exception of the catalytic inactive PARP13, catalyse MARylation [27]. Furthermore, the tRNA phosphotransferase TRPT1 (KptA in bacteria/Tpt1 in yeast) was identified as a highly divergent PARP family member that is conserved over all domains of life and catalyses tRNA dephosphorylation [39,40]. Finally, the sirtuin family, that is evolutionary unrelated to ARTs, contains also members capable of catalysing an ADP-ribose transfer reaction, by acting usually as protein deacetylases of lysines producing an *O*-acetyl-ADP-ribose (*O*AADPr) molecule [41] or less frequently by ADP-ribosylating proteins [42].

The ART fold is structurally highly conserved and binds NAD^+^ in a bent and constrained confirmation. Characteristic for the ART fold are mainly two known conserved three amino acid motifs that are critical for NAD^+^ binding and catalysis [43]. The identity of the motif, either [H-Y-E] present in ARTDs or [R-S-E] in ARTCs (including variants of both motifs), allows classification of ARTs to those respective subfamilies [36,44]. Both motifs have a glutamate in common which is a key residue for catalysing the ADP-ribosylation reaction and ART variants lacking this acidic residue itself substitute it from their substrate through a process termed substrate-assisted catalysis [45]. The ADP-ribosylation reaction generally proceeds via an S_N_1 mechanism which is characterised by NAD^+^ cleavage, generating a reactive oxocarbenium ion as an intermediate for nucleophilic attack of the targeted acceptor. This is accompanied by anomeric inversion of the carbon C1 of the adenine-distal ribose and leaving of NAM as a reaction by-product (Figure 1) [44,46,47].

**Figure 1.**
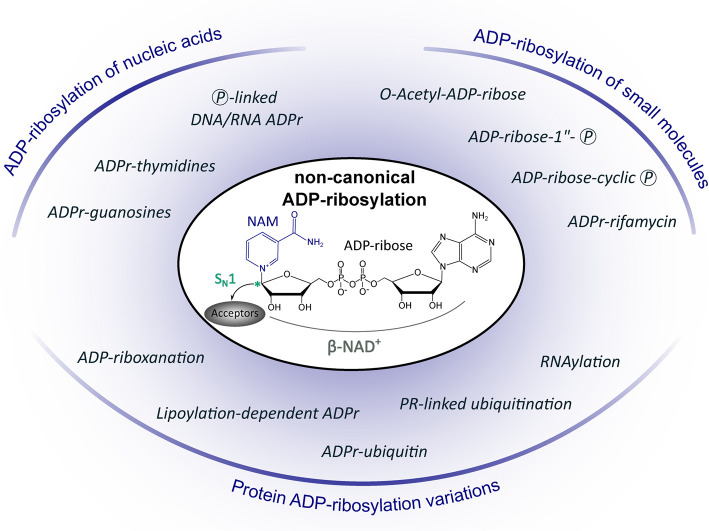
Overview of non-canonical ADP-ribosylation reactions discussed in this review. (ADPr: ADP-ribosylated/ADP-ribosylation; NAM: Nicotinamide; Ⓟ: phosphate; PR: phosphoribosyl; S_N_1: Nucleophilic Substitution, First Order — reaction mechanism for catalysing ADP-ribosylation which involves an oxocarbenium ion generated by NAD^+^ cleavage for nucleophilic attack of acceptors; * anomeric carbon linking ADP-ribose to acceptors).

Reversal of ADP-ribosylation is provided by enzymes capable of cleaving the ADP-ribose modifications from the respective targets. Two evolutionary unrelated protein families are characterised to catalyse the reversal of the canonical protein ADP-ribosylation, i.e. macrodomain-containing enzymes (e.g. PARG, MacroD1/2, TARG1) and the ADP-ribosylhydrolases (e.g. ARH1, ARH3), which show differences regarding target residue specificities and activities [35,48]. PARG efficiently degrades PAR chains, however, is unable to remove the terminal protein-linked ADP-ribose unit [49,50]. This is taken over by MARylation-reversing hydrolases, whereby TARG1, MacroD1/2 show strong activity on acidic residues [51,52], ARH1 reverses arginine ADP-ribosylation [53,54] and ARH3 specifically removes serine-linked ADP-ribose modifications [55,56]. Furthermore, removal of ADP-ribosylation was seen to be catalysed by the NUDIX family member NUDT16 as well as ENPP1 which both cleave the ADP-ribose pyrophosphate bond, thus remove AMP and consequently leave a phosphoribosyl moiety on the target protein [57,58].

The concerted action and interplay of ARTs and hydrolases on a specific target residues is mechanistically best understood for serine-linked ADP-ribosylation in the context of DNA damage response [59]. For this, PARP1/2 are essential but not sufficient as it requires HPF1 as accessory factor to direct the ADP-ribosylation of serine and the synthesis of longer or shorter PAR chains [32,60,61]. By forming a composite active site with HPF1, PARP1/2 substrate specificity is switched from acidic residues towards serine residues on target substrates [62], whereby the PARP2/HPF1 complex was shown to bridge two DSBs in a conformation compatible with DNA ligation after double-strand breaks [63]. Reversal of serine ADP-ribosylation is controlled by the PAR-degrading PARG [50,64] followed by removal of the remaining ADP-ribose unit by ARH3 [55,56,60]. This way, serine ADP-ribosylation signalling is regulated in a tight and timely manner and with it the numerous associated processes utilising it for preserving genome stability [32,65].

Research on ADP-ribosylation signalling with its players is moving fast and expanding the last years owing to new methodologies and tools which are able to uncover new aspects including substrate targets, catalytic mechanisms of ARTs and hydrolases, as well as the cross-talk and combined signalling of ADP-ribosylation with other modifications such as ubiquitination. With in particular increasingly more ADP-ribosylation reactions and products uncovered that are seen as ‘atypical' or ‘special cases', it yet becomes clear that the ADP-ribosylation mark is far more than a protein PTM in the classical sense. This prompts to rethink the traditional view on ADP-ribosylation and with this review, we use the opportunity to provide an overview of those non-canonical ADP-ribosylation reactions (Figure 1).

## ADP-ribosylation of nucleic acids

### ADP-ribosylation of guanosines

**Figure 2.**
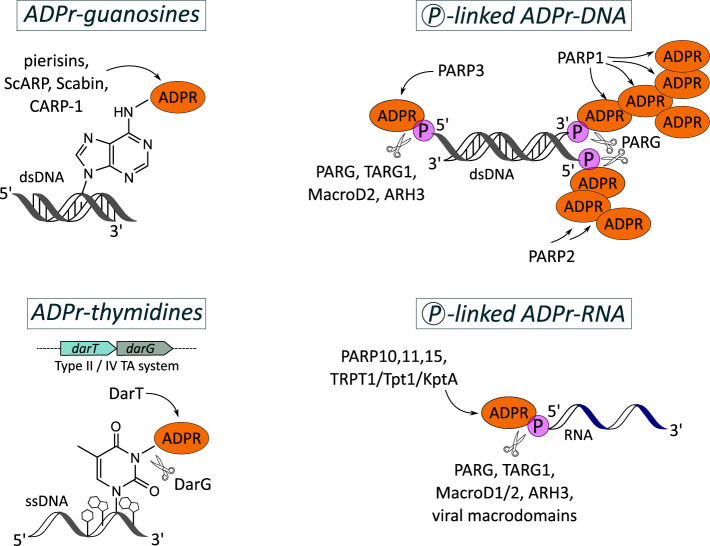
ADP-ribosylation of nucleic acids. (Top, left) Irreversible mono-ADP-ribosylation of guanosine bases by pierisins, ScARP, Scabin and CARP-1. (Bottom, left) Mono-ADP-ribosylation of thymidine bases in ssDNA by the toxin DarT which is reversible by its antitoxin partner, DarG. (Top, right) Mono- and poly-ADP-ribosylation of the DNA backbone via terminal phosphate-linkage catalysed by PARP1–3. The modifications are reversible by macrodomain-containing proteins (PARG, TARG1, MacroD2) and ARH3. (Bottom, right) Mono-ADP-ribosylation of the RNA backbone via phosphate-linkage catalysed by PARP family members and TRPT1/TPT1/KptA. (ADPR: ADP-ribose; ADPr: ADP-ribosylated; Ⓟ: phosphate; TA: Toxin-Antitoxin system).

One of the first non-canonical ADP-ribosylation of guanosines reaction was described with the discovery of the toxin pierisin-1 isolated from cabbage butterfly larvae, *Pieris rapae*, that showed mono-ADP-ribosylation activity specifically on nucleic acids instead on protein targets [66]. More pierisin family members (pierisin-1–5, ScARP, Scabin) and related proteins including CARP-1 from shellfish species were identified which all were characterised to belong to the ARTC subclass and to attach ADP-ribose to the N^2^ amino group of guanosine bases in either dsDNA, ssDNA or guanine-derived nucleosides (Figure 2, top left panel) [67–70]. Thus, although the same guanine specificity is shared, the pierisin-like enzymes show differences in substrate preference and their reaction has also a relaxed specificity regarding the target DNA motif. The mechanism of the enzymatic activity and the substrate recognition of pierisin-like ARTs has been best understood on the models of pierisin-1, ScARP and Scabin [68,70,71]. Studies on piersin-1 revealed that the activity of its N-terminal catalytic domain is controlled (in contrast with ScARP and Scabin) by an autoinhibitory linker that connects to the C-terminal ricin B-like domains. The latter enable binding to surface glycosphingolipid receptors for internalisation into cells. Following its incorporation into lysozymes, pierisin-1 is cleaved which releases the catalytic cleft-occupying linker region and consequently activates the enzyme. In this form, pierisin-1 is released into the cytosol and migrates into the nucleus to target genomic DNA for ADP-ribosylation [71]. Furthermore, a complex structure of ScARP with NADH and GDP provided details regarding the target recognition by the ART-conserved substrate binding region, the ADP-ribosylating turn-turn (ARTT) [68]. And finally, complementing kinetic studies of the DNA ADP-ribosylation reaction were performed with Scabin, a toxin secreted from the plant pathogen *Streptomyces scabies*, that shows preference for modifying dsDNA with a single-base overhang on either terminus, i.e. nicked dsDNA substrates [70].

So far, the ADP-ribosylation reaction catalysed by pierisin-like ART members is understood for being irreversible which induces strong genotoxicity and cytotoxicity including in several human cancer cell lines, making such toxins likely to be utilised as protective agents against microbes, viruses or (in the case of butterflies) parasitic wasps [72,73]. Yet apart from the natural purpose of these toxins, in an application-oriented study silkworms were bioengineered to express a less toxic variant of piersin-1 which instead of inducing apoptosis resulted in the dysfunction of their silk glands. The modified silkworms consequently produced cocoons without the silk protein fibroin and solely with the glue-like glycoprotein sericin which could be used as new biomedical material e.g. for tissue engineering [74].

### ADP-ribosylation of thymidines

The ARTC-class pierisin family members are not the only ARTs found to be capable of ADP-ribosylation of DNA bases. In 2016, a bacterial ARTD family member phylogenetically related to the eukaryotic PARPs was identified that catalyses the mono-ADP-ribosylation of thymidines (Figure 2, bottom left panel) and therefore named based on its general enzymatic activity DNA-ADP-ribosyltransferase, DarT [75]. In contrast with pierisins, the ADP-ribosylation reaction by DarT is characterised through target specificity and reversibility. Thus, DarT specifically modifies thymidine bases present in ssDNA, thereby showing no activity on other macro-biomolecules such as dsDNA, RNA or proteins. Its sequence specificity somewhat varies among different bacterial species, yet a four-base motif can be generalised for all DarTs with flexibility in all nucleotide positions apart from the third as being the thymidine that is targeted for ADP-ribosylation. As example, DarT of *Thermus aquaticus* and enteropathogenic *E. coli* (EPEC), which were among the first biochemically characterised variants, show a motif preference for TNTC and TTT/TCT, respectively (the underlined T is ADP-ribosylated) [75,76]. Only recently, mechanistic studies on *Thermus* sp. 2.9 revealed that DarT links ADP-ribose with the anomeric carbon of the adenine-distal ribose to the in-ring nitrogen of the thymidine base. The reaction requires an additional catalytic arginine residue in the active site that is assumed to be particularly essential for proton abstraction from the thymine nitrogen and that extends the canonical set of so far known ART catalytic residues [77]. It is indicative for the evolving diversity of ARTs with their ability to adapt to versatile specialised functions unrelated to the classical protein-targeting activity. The DarT-catalysed reaction can be reversed by the macrodomain-containing hydrolase DarG (DNA ADP-ribosylglycohydrolase) acting, compared with DarT, in a motif sequence-independent manner, thus is also able to reverse DarT reactions from non-cognate species [75,77]. By being downstream-coded in the same operon, DarG is genetically linked to DarT and together, DarT and DarG (DarTG) form a toxin-antitoxin (TA) pair. The DarTG TA system was the first to be discovered utilising ADP-ribosylation and is found in many bacterial including pathogenic species such as *Mycobacterium tuberculosis*, *Pseudomonas aeruginosa*, *Acinetobacter baumannii* and EPEC [75]. As the toxin of the system, DarT induces strong bacteriostatic effects and activates the SOS-response since the thymidine-linked ADP-ribosylation modifications are perceived as severe DNA damage requiring two consecutive DNA-repair pathways (HR and NER) to be resolved in a DarG-independent manner [75,76]. DarT expression was also shown to exert highly toxic effects in human cells which can however be protected by the endogenous human TARG1 enzymatic activity [78]. TARG1 displays close structural homology to DarG sharing the same catalytic lysine residue in the active site [52] and was found, like DarG, to be able to reverse thymidine-linked DNA ADP-ribosylation. Consequently, the expression of bacterial DarT toxin in TARG1-deficient human cells causes extreme replication stress impacting replication fork progression and activating the DNA-damage response at DNA replication sites [78]. For bacteria, DarG — that is functioning as the antitoxin — is an essential gene for survival and relevant for enabling smooth replication processes in DarT-expressing cells by providing DarT control and regulation via the hydrolytic activity of its macrodomain as well as physical sequestration through complex formation [75,76]. The relevance of DarG for bacterial survival and growth has been started to be understood in mycobacteria in which DarTG is among the three (out of ∼80 putative) TA systems that encode an essential antitoxin as demonstrated by transposon mutagenesis studies [79]. Mechanistically, mycobacterial DarT was shown to specifically ADP-ribosylate TTTW sequences that are abundant at the origin of chromosome replication (OriC). These OriC ADP-ribose modifications are assumed to impair the loading and DNA unwinding activity of the main replicative helicase DnaB (which is furthermore transcriptionally linked to DarTG), resulting in the phenotypic control of bacterial growth [77]. It is noteworthy, that carefully controlled, slow and nonreplicating growth states are key for *M. tuberculosis*, resulting in persistent, potentially life-long infection and antibiotic tolerance. DarT activity could therefore be a strategy employed by bacteria to induce persistence, i.e. a dormancy-like state that has been involved in gaining antibiotic resistance [75,80]. The latter was also seen as a result of increased mutability of mycobacterial strains upon experimental depletion of DarG [81]. Thus, it seems conclusive that DNA base ADP-ribosylation is used not only for targeted DNA damage to induce host-protective toxicity but instead is a far more complex signalling strategy for regulating bacterial physiology and pathogenicity. The observation that in pathogenic *E. coli* and cyanobacteria DarTG is often found to be inserted in an operon structure containing a type I restriction modification system suggested a link of DarTG to antiphage response and immunity [75,76]. Indeed, recently a role of DarT in antiphage defence was uncovered, where DarT is thought to modify invading viral DNA, thereby preventing its replication and consequently the production of virions, as a mechanism to control phage infections [82].

### Phosphate-linked DNA/RNA ADP-ribosylation

While the ADP-ribosylation of DNA bases requires specific recognition of the nucleotide by the ART, a more general way for DNA ADP-ribosylation signalling is the attachment of ADP-ribose to the nucleic acid backbone — a reaction that is catalysed by different PARP family members (Figure 2, top right panel) [83]. Corresponding to their observed ADP-ribosylation activity on protein substrates, PARP1 and PARP2 were shown to directly PARylate the terminal phosphate ends of DNA whereas PARP3 was characterised for MARylation of the latter [84]. The enzymes thereby show slight differences regarding their substrate preferences: For PARP1, the 3′-terminal phosphate at dsDNA break (blunt) ends on gapped DNA duplexes were found as major acceptor sites [85], while PARP2/3 preferentially target the 5′-terminal phosphate at blunt ends of nicked dsDNA [84,86]. The 5′-phosphorylated DNA breaks, which are recognised via DNA-binding domains, are thought to function as allosteric activators of the ADP-ribosylation activity of PARP1-3 by inducing structural alterations in the catalytic domain and thus relieving the autoinhibitory state [87]. The tRNA 2′-phosphotransferase 1 TRPT1/Tpt1/KptA also targets the 5′-phosphorylated ends for ADP-ribosylation, yet of ssDNA, which was shown as conserved Tpt1 activity among pro-/eukaryotic and archaeal species [88,89]. The DNA mono-ADP-ribosylation reactions catalysed by PARPs and TRPT1/Tpt1/KptA were all shown to be reversible by hydrolases including PARG, MacroD2, TARG1 and ARH3 [84]. Although these phosphate-linked ADP-ribosylation products were so far only observed *in vitro*, there is strong indication for their *in vivo* existence and thus physiological relevance [86]. The substrates used for testing PARP DNA ADP-ribosylation activity represent different types of DNA damage, hence allow to speculate about potential roles of this modification in the respective DNA repair pathways. For example, ADP-ribosylation at DNA ends could facilitate recruitment of DNA repair factors, protect DNA ends from unregulated nuclease activity [84] or serve as substrate for DNA ligases facilitating dsDNA ligation [90]. Finally, it is also hypothesised that this type of DNA ADP-ribosylation may be an erroneous activity of PARPs leaving ADP-ribose-DNA adducts similar to DNA adenylates formed upon abortive DNA ligations which are then repaired by PARG in a non-canonical DNA repair reaction and similar to aprataxin [84,91].

Similarly to DNA, the 5′-phosphate termini of RNA ends are targeted for MARylation by the human PARP10, 11, 15 and TRPT1 [88], as well as by the TRPT1/Tpt1/KptA homologues from lower organisms (Figure 2, bottom right panel) [88,89]. RNA ADP-ribosylation is also a reversible process through hydrolytic activity of PARG, TARG1, MacroD1/2 and ARH3 [88]. Interestingly, phosphate-linked RNA MARylation catalysed by PARP10 can also be reversed by viral macrodomains including the ones encoded by coronaviruses [88] and directly links this modification to potential functions in an immunity-related context. PARP10 is known to be induced by interferons, to regulate NF-κB signalling and to have inhibitory effects on viral replication including the positive-sense RNA alphavirus VEEV [92–94]. The RNA-recognizing motif (RRM) domains in PARP10 may be responsible to differentiate host from invading viral RNA on which the ADP-ribose modification is then terminally attached by the PARP10 catalytic domain. The phosphate-linked ADP-ribosylation is hypothesised to act as a non-canonical RNA cap preventing viral RNA translation or triggering signal transduction [88] while the viral macrodomain evolved to counteract this antiviral response. More generally, terminal RNA ADP-ribosylation may be seen as non-canonical RNA capping providing RNA stability against nuclease-mediated degradation [88] or involved in RNA signal transduction [95].

In conclusion, over the last years ADP-ribosylation of nucleic acids was established as a function of well-known and newly identified ARTs and will find its relevance in different physiological contexts next to protein ADP-ribosylation. Genomic data provides evidence that DNA ADP-ribosylating enzyme systems are conserved in a variety of organisms of all domains of life and the evolution of specific enzymatic proteins to guarantee the reversibility of the reaction (i.e. transferases/hydrolases) further underlines that nucleic acid ADP-ribosylation likely presents a wide-spread form of ADP-ribosylation signalling. The current technical challenges regarding its detection and tracing are about to be overcome which will shed light on this so far largely unexplored facet of ART research.

## ADP-ribosylation of small molecules

**Figure 3.**
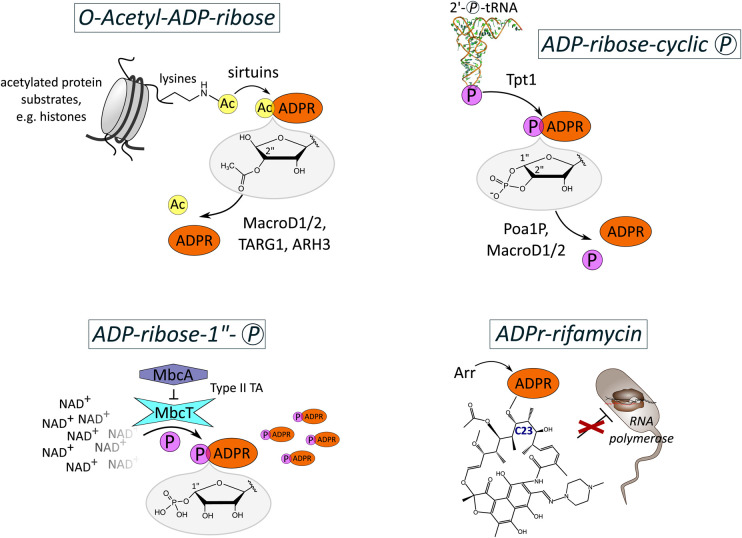
ADP-ribosylation of small molecules. (Top, right) De-acetylation of proteins by sirtuins results in generation of *O*-acetyl-ADP-ribose which is cleaved to ADP-ribose and acetate by the hydrolases MacroD1/2, TARG1 and ARH3. (Top, left) De-phosphorylation of tRNA as intermediate step in tRNA splicing by Tpt1 family members releases mature tRNA and ADP-ribose-cyclic phosphate. The latter is cleaved to ADP-ribose and phosphate by the hydrolases Poa1P and MacroD1/2. (Bottom, left) The toxin MbcT of the type II TA system MbcTA functions as a NAD^+^ phosphorylase, thereby generating ADP-ribose-1″-phosphate. Binding of the antitoxin MbcA to MbcT inhibits toxin activity that is stimulated by inorganic phosphate. (Bottom, right) Mono-ADP-ribosylation of rifamycin by Arr enzymes leads to loss of the antibiotic activity of the molecule by inhibiting the binding of rifamycin to its target, i.e. the DNA-dependent RNA polymerase. (Ac: Acetate; ADPR: ADP-ribose; Ⓟ: phosphate; TA: Toxin-Antitoxin system).

Several classes of enzymes are known to produce ADP-ribosylated small chemical molecules as by-products of their activities (Figure 3). Among these are the sirtuins that produce *O*AADPr by catalysing the deacetylation of lysine residues from proteins including histones for the regulation of processes in all kingdoms of life (Figure 3, top left panel) [96–98]. In contrast with histone deacetylases (HDACs) and HDAC-related enzymes that utilise an active-site metal ion (Zn^2+^ or Fe^2+^) to direct a water-mediated attack hydrolysing the acetyl-lysine residue [99], the deacetylation activity of sirtuins is an NAD^+^-dependent catalysis, releasing nicotinamide, deacetylated lysine and *O*AADPr in a multi-step reaction [100,101]. *O*AADPr may moreover act as an important signalling molecule itself with functions implicated in TRPM2 cation channel gating, modulation of the cellular redox status as well as gene silencing by facilitating the loading of the Sir2–4 silencing complex onto nucleosomes [102,103]. Its binding to the macrodomain of the histone variant macroH2A1.1 might furthermore be relevant for inducing macroH2A1.1-dependent chromatin changes [104]. Other macrodomain-containing proteins, i.e. MacroD1/2 and TARG1, along with ARH3 are also able to recognise this NAD^+^ metabolite, and hydrolyse it to ADP-ribose and acetate [105–107]. The relevance of MacroD protein in *O*AADPr hydrolysis was confirmed *in vivo* using the filamentous fungus *Neurospora crassa* as model organism, in which deletion of MacroD led to a notable increase in *O*AADPr levels [105].

The highly conserved Tpt1 family presents another class of enzymes that produces ADP-ribosylated signalling molecules by transferring a single ADP-ribose unit to terminal 2′-phosphates of tRNA which is an intermediate step in the tRNA splicing process in plants, fungi and yeasts [39]. The ADP-ribosylation reaction is followed by non-enzymatic generation of ADP-ribose-cyclic phosphate, releasing the mature tRNA (Figure 3, top right panel) [108]. Since prokaryotes and other eukaryotes seem to lack intron-containing tRNAs requiring splicing, these Tpt1 homologues likely exert their functions as phosphotransferases on alternative targets, including through their ADP-ribosylation activity on 5′-phosphorylated RNA ends as mentioned above. Although it remains to be clarified whether ADP-ribose-cyclic phosphate has itself a signalling function, it is known to be hydrolysed to ADP-ribose by yeast Poa1P protein and other homologous macrodomain proteins including MacroD1/2 [109,110].

Recently, a novel type II TA system, referred to as MbcTA, was identified that is found in multiple mycobacterial species and that encodes a toxin, MbcT, showing structural similarity to ARTs as well as NADases [111]. MbcT was characterised as a NAD^+^ phosphorylase generating ADP-ribose-1″-phosphate by catalysing the transfer of ADP-ribose from NAD^+^ onto inorganic phosphates, whereby the latter functions as stimulator for the reaction itself. In the absence of the antitoxin MbcA, MbcT activity was found to result in depletion of intracellular NAD^+^ which triggered rapid cell death in *M. tuberculosis*, consequently prolonging the survival of infected mice (Figure 3, bottom left panel) [111].

Finally, some ARTs are known to have evolved in bacteria as a defence mechanism against viruses, other bacterial species and also including antimicrobial molecules [44]. Among these is the Arr-ms family, named after the rifampin ADP-ribosyltransferases (Arr), whose members belong to the ARTD-class. They were identified in mycobacterial species as well as gram-negative pathogenic bacteria, that catalyse the ADP-ribosylation of rifamycin antibiotics as a strategy to confer antibiotic resistance for the bacterium [112,113]. The antibacterial action of rifamycins is based on their inhibition of the DNA-dependent RNA polymerase through binding with high affinity [114]. Arr enzymes attach the ADP-ribose unit to the hydroxyl moiety on carbon 23 which interacts with the amide backbone of the RNA polymerase. ADP-ribosylation thus interrupts this interaction and leads to inactivation of the antibiotic (Figure 3, bottom right panel) [112,114]. The observations by Baysarowich et al. [112] that Arr enzymes show broad substrate specificity with similar kinetic constants and that specific interactions between Arr and rifampin are absent from in the co-crystal structure suggests that these ARTs likely have other cellular functions including possibly the ADP-ribosylation of other small molecules.

## Non-canonical protein ADP-ribosylation

**Figure 4.**
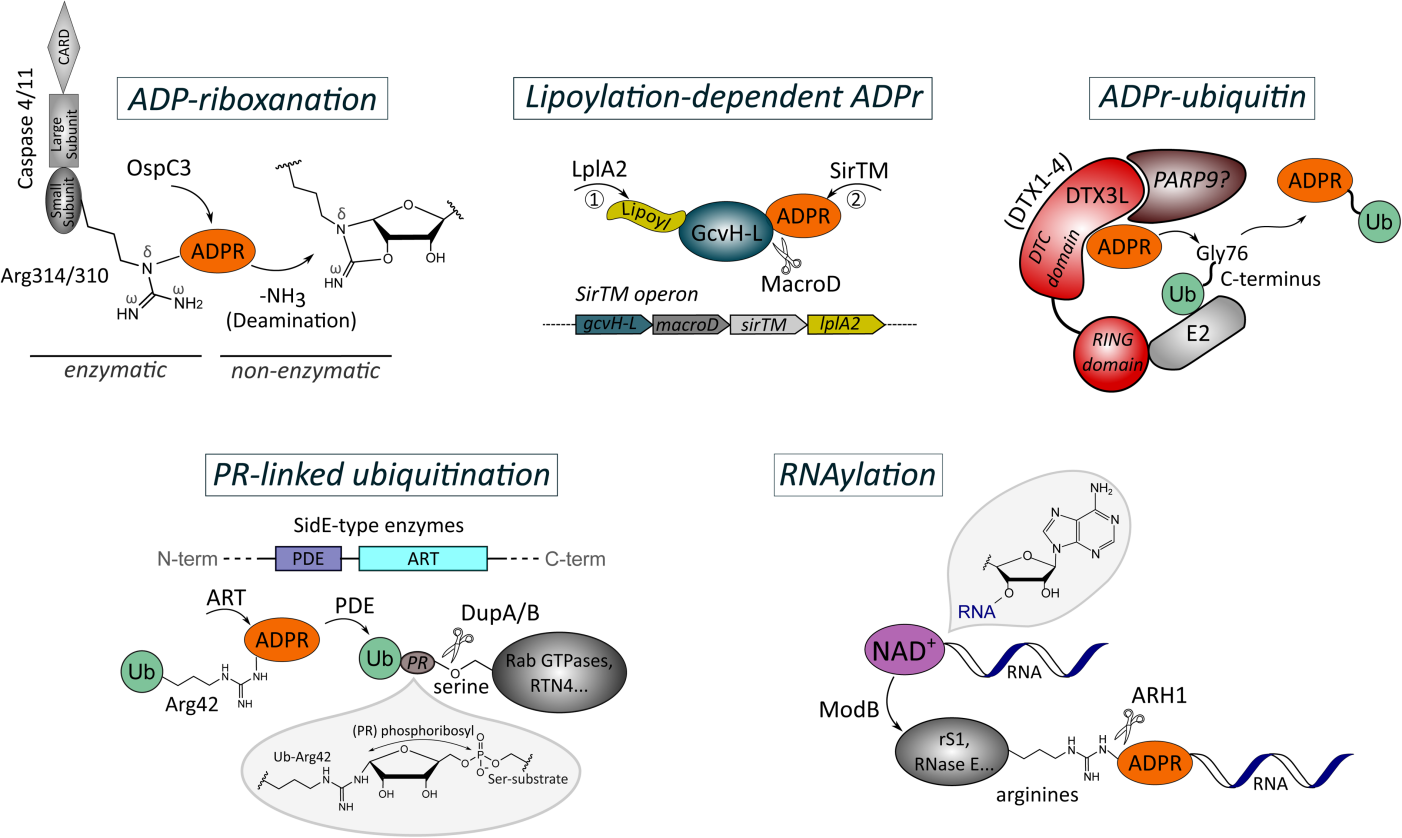
Non-canonical protein ADP-ribosylation. (Top, left) The type III secretion system effector of *Shigella flexneri*, OspC3, ADP-ribosylates the caspase-conserved arginine residues in caspase 4/11 (Arg314/Arg310) which is followed by non-enzymatic internal deamination — a process termed ADP-riboxanation. The modification is understood to be irreversible and to provide means for the pathogen to escape the inflammatory response of the host. (Top, middle) In *Staphylococcus aureus* and *Streptococcus pyogenes*, ADP-ribosylation activity of SirTM is dependent on prior lipoylation of its specific target, the lipoyl-carrier protein GcvH-L, by the lipoate-protein ligase A (LplA2). The modification is reversed by a MacroD hydrolyse which is encoded within the same operon as SirTM, GcvH-L and LplA2. (Top, right) The DTC domain of DTX1-4 ADP-ribosylates the C-terminus of ubiquitin at Gly76 which is recruited to site of action by the interaction of the RING domain of DTX1–4 with E2 ligase. PARP9 forms a complex with the family member DTX3L, yet its precise role in this ADP-ribosylation reaction is still unclear. (Bottom, left) In a two-step reaction, SidE-type bacterial effectors first ADP-ribosylate with their ART domain ubiquitin at Arg42. The pyrophosphate of the ADP-ribose modification is then cleaved by their PDE domain resulting in phosphoribosylated ubiquitin (PR-Ub) which is conjugated to serine residues in substrate proteins. The PR-UB modification can be removed from the serine residues by the hydrolases DupA/B. (Bottom, right) The ARTC-class member produced by T4 bacteriophage, ModB, attaches RNA chains to arginine residues of host acceptor proteins via a diphosphoriboside linkage by utilising NAD^+^-capped RNAs as substrate. The modification can be reversed by ARH1. Amino acid identifier refer to human proteins in the ADP-riboxanation, ADPr-ubiquitin and PR-linked ubiquitination panels. (ADPR: ADP-ribose; ADPr: ADP-ribosylation; ART: ADP-ribosyltransferase; DTC: ‘Deltex carboxyl-terminal’ domain; PDE: Phosphodiesterase; PR: phosphoribosyl; Ub: ubiquitin).

Besides the different substrates that can be targeted and modified by ARTs, ADP-ribosylation of proteins itself can be subjected to variations which are beyond its signalling forms as canonical target MARylation or PARylation (Figure 4). The ART-catalysed ADP-ribose modification can be derivatised on the target protein, providing a possibility to escape the reversibility of the reaction through common hydrolases and making it a more persistent signal [115]. Furthermore, ADP-ribosylation activity and substrate specificity can be additionally regulated by being dependent on prior target modification by other PTMs [42]. Some of the latter are known to cross-talk with ADP-ribosylation signalling which in case of ubiquitin as protein PTM includes the possibility to be a target for ADP-ribosylation itself and thus, to combine PTM signals [116,117]. Moreover, ADP-ribosylation can function as a linker enabling the attachment of macromolecules to the target substrate [118–122].

### ADP-riboxanation

The human pathogen *Shigella flexneri* is among the few bacterial species living freely in the host cytosol, thus inevitably exposing its lipopolysaccharides (LPS) to the inflammasome and inflammatory caspases [123]. It evolved therefore a virulence mechanism that prevents LPS-induced pyroptosis that is mediated by caspase-11 or caspase-4 using a type III secretion system (T3SS) effector, OspC3 [115]. OspC3 targets caspase-conserved arginine residues in caspase 4/11 (Arg314/Arg310) by ADP-ribosylation. Yet instead of transferring the ADP-ribose unit in a canonical manner to an arginine *N*^ω^ nitrogen, OspC3 first ADP-ribosylates the *N*^δ^ nitrogen which is followed by a (non-enzymatic) internal deamination initiated by the 2″-hydroxyl group of the ADP-ribose to remove one *N*^ω^ nitrogen, thereby forming an oxazolidine ring. The OspC3-catalysed reaction was therefore termed ‘ADP-riboxanation’ (Figure 4, top left panel). Due to its non-canonical arginine linkage and ribose modification compared with arginine ADP-ribosylation, Li et al. [115] found that ADP-riboxanation cannot be hydrolysed by the ADP-ribosylhydrolase ARH1, which catalyses canonical ADP-ribosyl-arginine linkages, or other host hydrolases including ARH3, TARG1 and MacroD1/2. This makes ADP-riboxanation of caspase-4/11 more pathogenically advantageous which was shown to block caspase activation and cleavage of their substrate, the pore-forming protein GSDMD, due to structural interference with the GSDMD-binding exosite [115].

### Lipoylation-dependent ADP-ribosylation

A distinct class of sirtuins referred to as ‘SirTMs’ and predominantly identified in bacterial and fungal pathogens was shown to lack the characteristic protein deacetylase activity of sirtuins and instead reliably catalyses the ADP-ribosylation of proteins [42]. In *Staphylococcus aureus* and *Streptococcus pyogenes*, this SirTM activity was furthermore dependent on prior lipoylation of its specific target, the lipoyl-carrier protein GcvH-L, by the lipoate-protein ligase A, LplA2. GcvH-L and LplA2 are encoded together with SirTM within the same operon which also includes a macrodomain protein, termed MacroD, that is capable of reversing GcvH-L ADP-ribosylation — in contrast with its homologues in humans (MacroD1) and *E. coli* (YmdB) (Figure 4, top middle panel) [42,124]. These operon-specific activities showing a cross-talk between lipoylation (probably acting as scavenger of reactive oxygen species) and MARylation (thought for guiding protein interactions) were suggested to be implicated in regulating oxidative stress response in these pathogens, thus providing a host defence mechanism [42].

### ADP-ribosylation of ubiquitin

Over the last years, there have been growing examples of the interplay between ADP-ribosylation and ubiquitination. Ubiquitination is typically catalysed by the three-enzyme cascade involving ubiquitin-activating enzymes (E1s), ubiquitin-conjugating enzymes (E2s), and ubiquitin ligase enzymes (E3s), which attach the carboxy terminus of ubiquitin to, in most cases, the ε-amino group of a substrate lysine via an isopeptide bond [125]. However, heterodimerization of the E3 ligase DTX3L with its co-expressed binding partner PARP9 was shown to instead result in ADP-ribosylation of the ubiquitin carboxy terminus; the attachment of ADP-ribose to the C-terminal glycine (Gly76) of ubiquitin not only interferes with its conjugation to substrates but also with its activation and transfer along the E1–E2–E3 cascade [116,126]. The ubiquitin ADP-ribosylation activity of the DTX3L/PARP9 complex is stimulated by PAR binding of the macrodomains of PARP9. This is analogous to the allosteric activation of the E3 ligase RNF146 through PAR binding of its WWE domain [127]. PARP9 was suggested to be a catalytically active ART, restraining the E3 function of DTX3L in the context of DNA repair [116], yet the exact mechanistic role of PARP9 in the ubiquitin ADP-ribosylation process is still unclear. Further mechanistic studies moreover revealed the conserved RING-DTC (‘Deltex carboxyl-terminal’) domains from DTX3L and other human Deltex family members (DTX1–4) as being able to catalyse the linkage between ubiquitin and ADP-ribose [117]. While the RING domain recruits the ubiquitin-loaded E2, the DTC domain binds the NAD^+^ substrate, whereby the linker between the RING and DTC domain facilitates the juxtaposition of both domains for the ADP-ribose transfer onto the carboxylate group of ubiquitin's glycine terminus (Figure 4, top right panel). Nonspecific deubiquitinases are able to recognise and reverse the ADP-ribosylation modification on ubiquitin, indicating a dynamic nature of this signal [117]. For DTX2, it was shown that it is predominantly associated with the DNA damage response and PARP1 through binding of PARylated DNA repair proteins [128].

### Phosphoribosyl-linked ubiquitination of proteins

A non-canonical type of protein ADP-ribosylation is utilised by bacterial effectors belonging to the SidE family (SdeA, SdeB, SdeC, and SidE) that are produced by *Legionella pneumophila*, the pathogen causing pneumonia infections known as Legionnaires’ disease [120,129,130]. SidE-type enzymes are characterised by the linkage of a phosphodiesterase (PDE) domain to an ART domain [122,131]. The combination of both active domains allows the enzymes to catalyse the conjugation of ubiquitin via a phosphoribosyl moiety to serine residues of host substrates (Figure 4, bottom left panel). Mechanistically, the ART domain (a member of the ARTC-class) first transfers ADP-ribose from NAD^+^ to the side chain of Arg42 on ubiquitin (Ub) to generate ADPr-Ub. This MARylation product is then recognised by the PDE domain which cleaves the ADP-ribose pyrophosphate bond resulting in phosphoribosylated ubiquitin (PR-Ub) that is then conjugated to serine residues in substrate proteins [118,121,122]. Hence, ADP-ribosylation is employed by SidE-type enzymes as an intermediate step to link ubiquitin (independently of E1 and E2 enzymes or ATP consumption) to their respective targets. The latter are several endoplasmic reticulum (ER)-associated human Rab GTPases and the ER protein reticulon 4 (RTN4) to control the dynamics of tubular ER for replication processes [129,132]. Reversal and thus regulation of the phosphoribosyl serine ubiquitination is achieved by DUPs (‘deubiquitinases for PR’), DupA and DupB, with specifically bind and cleave PR-Ub from the modified substrate serine [133]. PR-Ub itself has also a pathogenic function by inhibiting the host's conventional ubiquitination cascades and thereby impairing numerous cellular processes including mitophagy, TNF signalling, and proteasomal degradation [120].

### RNAylation

A subset of regulatory RNAs is found abundantly as NAD-RNA in *E. coli* [134]. Most recently, an ARTC-class member produced by the T4 bacteriophage, ModB, was characterised to catalyse the attachment of RNA chains to host acceptor proteins via a diphosphoriboside linkage by utilising NAD^+^-capped RNAs as substrate [119]. Thus in this case, ADP-ribosylation also functions as a linker between RNA and target protein, with the modification termed ‘RNAylation’ (Figure 4, bottom right panel). ModB was shown to RNAylate specific arginine residues of its targets, including the ribosomal protein S1 (rS1) and RNase E [119,135]. The RNAylation modification could be reversed by human ARH1 *in vitro* [119]. The role of these RNA-protein conjugates is so far unclear but was suggested to promote recruitment of phage mRNAs for ribosomal biosynthesis. Furthermore, RNAylation destabilises rS1 which could contribute to the shut-down of host mRNA translation during T4 infection and thus promote bacterial lysis [119,135,136].

## Conclusions

In summary, ADP-ribosylation displays a remarkable versatility. The molecular structure of the ADP-ribose unit provides several possibilities of linkages that allows ADP-ribosylation in its appearances as either monomeric or linear and branched polymeric form but also to be used for linking different biomolecules. Thereby, ARTs mainly direct — in some cases together with accessory factors — target specificity regarding the substrate type (proteins, nucleic acids, small molecules), the modification site and linkage to protein residue or nucleotide and the general form of ADP-ribosylation signal (MAR or PAR). So far, protein ADP-ribosylation is best-characterised and understood regarding its physiological relevance in various cellular processes. However, over the recent years, the ever more examples of non-canonical ADP-ribosylation reactions discovered extended the repertoire of this signalling strategy. These include new target substrates such as nucleic acids but also variations of the familiar protein ADP-ribosylation modification regarding cross-talk to other PTMs and inventive ways to modulate host-microbial interaction. Considering that ADP-ribosylation is a very ancient type of target-modifying signal with conservation among all domains of life, its versatility may have allowed to evolve many more non-canonical ADP-ribosylation-based modifications only waiting to be explored. The evolutionary spread may also indicate that these ADP-riboslyation reactions are more than just ‘special cases' or small ‘side reactions' which questions our description as being ‘non-canonical'. The heavy focus on ADP-ribosylation as protein modification may have just overshadowed the richness of ADP-ribosylation as general (protein-unrelated) signalling event. Its study will not only uncover exciting facets of life and evolution but also comes with great potential for the development of new antimicrobials and anticancer agents as well as biotechnological tools [73–75,77,78]

## Open Access Statement

Open access for this article was enabled by the participation of University of Oxford in an all-inclusive Read & Publish pilot with Portland Press and the Biochemical Society under a transformative agreement with JISC.

## Abbreviations

ADPR: ADP-ribose
ART: ADP-ribosyltransferase
ARTDs: ADP-ribosyltransferases
LPS: lipopolysaccharides
NAD^+^: nicotinamide adenine dinucleotide
*O*AADPr: *O*-acetyl-ADP-ribose
PAR: poly-ADP-ribose
PDE: phosphodiesterase
PTM: post-translational modification
TA: toxin-antitoxin
